# Five ways to wellbeing at the zoo: improving human health and connection to nature

**DOI:** 10.3389/fpsyg.2023.1258667

**Published:** 2023-09-21

**Authors:** Paul Rose, Lisa Riley

**Affiliations:** ^1^Centre for Research in Animal Behaviour, Psychology, University of Exeter, Exeter, United Kingdom; ^2^WWT, Slimbridge Wetland Centre, Slimbridge, United Kingdom; ^3^Centre for Animal Welfare, University of Winchester, Winchester, United Kingdom

**Keywords:** animal welfare, human wellbeing, nature connection, zoo, mental health

## Abstract

Good mental and physical health go hand-in-hand when identifying factors that lead people to experience a better overall quality of life. A growing disconnect to the natural world is worsening the mental health of individuals in many societies. Numerous scientific publications have evidenced that being in nature and access to green and blue spaces positively impact upon humans’ physical and mental health. For many people, particularly those living in more urbanized areas, managed natural spaces and borrowed landscapes, such as those found in public parks, wildlife reserves and zoological gardens give the only opportunities for wider engagement with nature. Many zoos are designated green spaces and therefore human visitors can engage with native fauna and flora as well as exotic wild animals. This article reviews the UK Government’s “The Five Ways to Wellbeing” concept, applied to zoos and aquariums and thus suggests how zoos and aquariums can use this framework to promote positive nature-connectivity experiences for their visitors and promote good wellbeing. The Five Ways to Wellbeing are Connect, Be active, Take notice, Keep learning, and Give. We illustrate how zoos and aquariums could model their approaches to educational and engagement roles, as well as design initiatives to reach out to local communities *via* the Five Ways to Wellbeing concept. We show that many of the positive programs and works conducted by zoos and aquariums lend themselves to further engagement with the Five Ways to Wellbeing structure. By taking such a structured approach in the design, implementation and evaluation of their activities, zoos can expand their abilities in connecting humans with nature and further add value to their living collections of animals and plants. By including Wellbeing as a defined aim of the modern zoo, it will be clear to all of those involved in their work, visitors, workers, stakeholders, that zoos are working to promote, protect and preserve positive wellbeing outputs for humans and animals alike.

## 1. Introduction

There is a global crisis around mental health (Patel et al., 2007; Tiwari, 2023), in part caused by contemporary challenges to living (Jakovljevic et al., 2020) modern ways of communicating and living (Kelly et al., 2018; Smith and Victor, 2019), and a widespread disconnection with nature and the natural world (Gelsthorpe, 2017). Access to nature has been shown to promote positive wellbeing and alleviate mild depression and anxiety in humans (Bratman et al., 2012; Keenan et al., 2021; Owens and Bunce, 2022; Irvine et al., 2023). As global populations continue to urbanize (United Nations, 2018), causing greater distance between centers of human habitation and wild environments (Cox et al., 2018), managed green and blue spaces (e.g., public parkland and gardens and nature-themed visitor attractions such as zoological collections) become more important to fostering a sense of “being in nature” (Baur and Tynon, 2010; Kellert, 2012; Arbuthnott et al., 2014; Taylor and Duram, 2021). Institutions that are ultimately centered on bringing nature closer to humans are zoological collections, such as zoos, aquariums, and safari parks (hereafter “zoos”). Although the number is hard to accurately define, there are an estimated 10,000 zoological collections globally (Glazier, 2017). A smaller proportion of this overall estimate will be part of accreditation (e.g., European Association of Zoos & Aquaria, EAZA; Association of Zoos & Aquariums, AZA) or membership (British & Irish Association of Zoos & Aquariums) organizations that uphold education, conservation and research initiatives and promote good animal welfare (Marcy, 2021; BIAZA, 2023a; EAZA, 2023a). Modern zoos are consistently aiming to promote both animal welfare and positive human wellbeing in terms of their outputs and operations (Rose and Riley, 2022) and therefore have value to the human populations that work at them, live around them, visit them and engage with their work on a local or global level (Greenwell et al., 2023). This value can be extended if zoo visits can enhance mental health, encourage a deeper understanding of nature, and foster a greater appreciation of the natural world.

The UK government defines the concept of human wellbeing as comprising of two main elements: feeling good and functioning well (CIEEM, 2021). This approach is similar to that outlined by the World Health Organization, who state that wellbeing is a positive state experienced by individuals and societies, that is important for daily life, and encompasses quality of life and the activities that people can get involved in World Health Organisation (2023). Therefore, enhancing opportunities to be outdoors with nature, and to engage with others whilst undertaking meaningful and fulfilling activities promotes these good feelings and positive physical and mental functions (Nisbet et al., 2011; Bratman et al., 2012; Cudworth and Lumber, 2021), which are the core of wellbeing.

**Figure 1:**
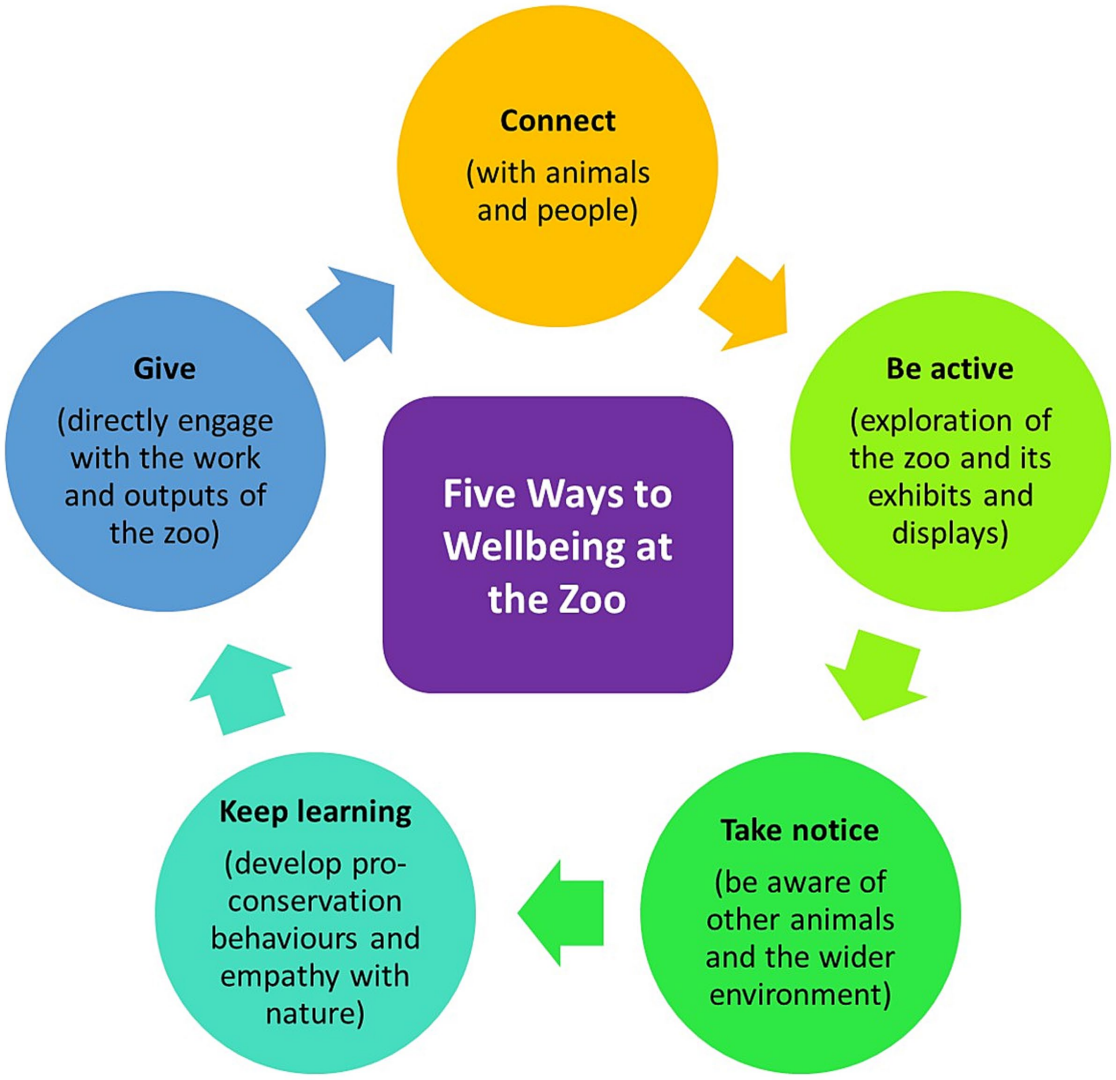
The Five Ways to Wellbeing as described by the New Economic Foundation with an example of how they integrate into a visit to a zoological collection.

An example of an approach to enhance human wellbeing to improve overall quality of life can be found in the Five Ways (or Steps) to Wellbeing that were published in 2008 by the New Economics Foundation on behalf of the UK Government (Aked et al., 2008b). This project was initiated to understand ways of promoting improvements to mental wellbeing in individual people and across society more widely, and of enhancing mental capital (i.e., a person’s cognitive and emotional resources). The Five Ways to Wellbeing are to Connect, Be active, Take notice, Keep learning, and Give (Aked et al., 2008b) and these are outlined in Figure 1. As a framework for evaluating human wellbeing, The Five Ways to Wellbeing have featured in several publications relating to human wellbeing and nature connectivity, including peer-reviewed research papers (Chiumento et al., 2018) and mainstream psychology publications (Harkness, 2019). And they have also been used within research, methodologies designed to measure good human wellbeing and improvements to quality of life, across numerous other disciplines in different parts of the world (Mahoney-Davies et al., 2017; Mackay et al., 2019; Gillard et al., 2021; Coren et al., 2022). The principles of the Five Ways to Wellbeing have been endorsed by the UK’s mental health charity, “Mind” (Mind, 2023) and are also widely advertised by the UK’s National Health Service as part of its mental health provision (National Health Service, 2022). Therefore, the Five Ways to Wellbeing approach is clearly seen as a credible formula for helping to provide practical support and tools to improve both individual and societal quality of life.

This article considers the key concepts of the Five Ways to Wellbeing and the role of zoos in providing meaningful connection to nature, opportunities to engage and interact with other individuals in a positive and constructive manner, and ways of getting involved in pro-conservation activities and initiatives. Promoting a connection to nature is essential if green and blue prescribing (nature-based interventions and activities prescribed to restore positive mental states, National Health Service, 2022) is to be wholly effective. It is centered in the concepts of green / blue prescribing that can offer treatment for mental health disorders, such as anxiety and depression (Owens and Bunce, 2022; Irvine et al., 2023) and that zoos could get involved with. This is the first time (to the authors’ knowledge) that zoos as potential sites to embed the Five Ways to Wellbeing has been explored. A literature search, conducted in June 2023 on Google Scholar^1^ and on Web of Science^2^ for the terms “Five Ways / Five Steps to Wellbeing zoo,” “Five Ways / Five Steps to Wellbeing aquarium,” “Five Ways / Five Steps to Wellbeing nature” revealed no articles to have employed this method to date in the context of zoo operations and aims. Therefore, our concept paper reviews the operational nature of zoos and their aims, in terms of each of the Five Ways to illustrate the potential for this approach to future investigation and research application. We provide examples of how zoos can engage with each one of the Fives Ways to Wellbeing “actions” (Connect, Be active, Take notice, Keep learning, Give) to maximize their positive impacts on human wellbeing and planetary health both locally and globally. We have explored the framework of the Five Ways to Wellbeing to show how the activities that zoos provide and promote fit within the ideals and aims of the Five Ways to Wellbeing regarding improvements to human quality of life.

## 2. Connect

Zoos provide spaces that enable people to connect; both with each other and with the natural environment. A key element of the Five Ways to Wellbeing is the building and maintenance of positive relationships with others as a crucial element for long-term well-being. In the zoo, connecting with family, friends, colleagues, and the wider community is possible and can provide feelings of belonging, support, and purpose. Zoo visits foster a sense of interest in nature, facilitate social support, and spark positive discussion on the animals that visitors interact with Clayton et al. (2009) and Clayton et al. (2014). As being connected fosters an individual’s sense of value and enhances social interactions (Martino et al., 2017), positive impacts on mental health and physical health become realized. Across the world, there are estimates that over 700 million people may visit a zoo annually (Gusset and Dick, 2011). By connecting individuals together as well as connecting people with nature, zoos can positively impact human health, and spread positive human Behavior change messages more widely that can ultimately benefit planetary health too (Falk et al., 2007; Jensen et al., 2017; Godinez and Fernandez, 2019).

Use of various social media platforms and engagement with online audiences can foster interest and attention in a specific theme or idea (Derby, 2013). The use of multiple social media platforms is beneficial for zoos to connect with wider audiences, especially with people who may not consider visiting a zoo or who may not have an immediate, deeper interest in animals and the natural world. Using social media platforms as a bridge between any potential interest in animals (at the zoo) and then going to visit such animals can encourage nature connectivity during a physical visit to the zoo itself. In this scenario, engagement with a social media platform sparks the interest that results in a visit to see animals at the zoo. For example, by presenting information on conservation and biodiversity in a factually correct yet accessible and entertaining manner, e.g., on YouTube (Llewellyn and Rose, 2021) or on Facebook (Rose et al., 2018) zoos can build links to under-represented groups that may not have originally considered visiting an animal collection or natural space.

**Figure 2:**
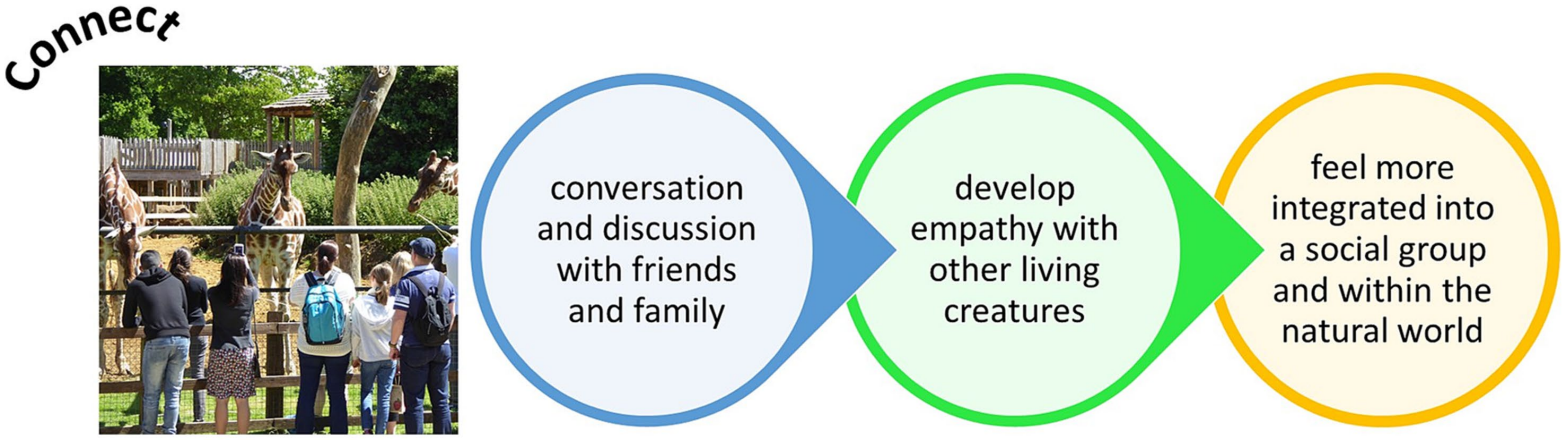
Ultimate benefits of being connected at the zoo and on a zoo visit.

Zoos should also broaden their audiences to reduce any perception that they are just places for families and children to have a fun day out in Esson and Moss (2014) and Turley (2001). As an emphasis on being a playground for children can deter others from visiting zoos (Turley, 2001), wider consideration of how to connect with a more diverse array of audiences would provide more personal value to a zoo visit and give wider impact of any mental and physical health benefits. As zoo visits provide people with opportunities to develop emotional connections with non-human animals (Clayton et al., 2009; Howell et al., 2019), there is the chance to encourage pro-conservation and sustainability Behavior change post-zoo visit. By seeing animals in close proximity, zoos help foster a bond between the visitor and the natural wonder (Vining, 2003) and this emotional connection may help foster compassion for and interest in wildlife, biodiversity and the health of the planet. As many people visit zoos as a group (e.g., in a family setting), such a connection to nature can spread across generations and be a talking point or topic of discussion and dialog between these individuals in their social group (Figure 2).

In the UK, zoos employ approximately 3,000 full-time staff (Animal Careers Direct, 2023) and in the United States, AZA accredited zoos employ 198,000 people (Marcy, 2021). Not only does this represent vast opportunity for connection, both AZA and BIAZA (and many zoo member and accreditation organizations globally) host annual conferences and have active working groups offering further, wider connectivity to like-minded people who share an interest in animals. The sharing of joint goals like achieving husbandry development and striving for improved animal welfare, therefore affords a sense of camaraderie and togetherness. Those staff who work directly with animals can also participate in stable, strong attachments with the animals in their care. With companion animals, such attachments are considered a positive human-animal interaction that is important for both good animal welfare and positive human wellbeing (Walsh, 2009). Melfi et al. (2022) found that zookeepers did form such attachments to animal in their care, although not as strongly as with their own companion (pet) animals. This research identified that female zookeepers were significantly more attached to zoo animals than male zookeepers and thus there is the potential for all zookeepers but especially females to connect with the animals in their care. Even those whose work does not directly involve animals have daily opportunities to interact with animals as they journey around the zoo throughout the working day. Thus, zoo staff have many opportunities for connectivity with human and non-human animals alike.

Zoos need to consider animal welfare states and how these are upheld and promoted to visitors (Sayers, 2020), especially when connecting visitors with nature. Promoting good animal welfare is likely to leave a lasting positive impression on zoo visitors as research has identified that when zoo visitors view abnormal Behaviors (e.g., stereotypic pacing), they leave with a poorer impression of the zoo overall (Miller, 2012). Negative impressions of captive wildlife can be caused by a visitor’s experiences of poor animal management (Woods, 2002), thus detracting from the zoo’s value and its ability to connect more deeply with the audiences that visit. Likewise, the behavior of visitors themselves can disturb the animals themselves and create a negative atmosphere at the zoo (Collins et al., 2023), preventing others’ attempts at connecting with nature more widely, or animals specifically, in the zoo. Consequently, zoos need to actively manage visitor Behavior, engaging with them to eliminate negative actions that compromise animal welfare and the experiences of other visitors who wish to fully connect with nature during their time in the zoo’s living collection.

## 3. Be active

**Figure 3:**
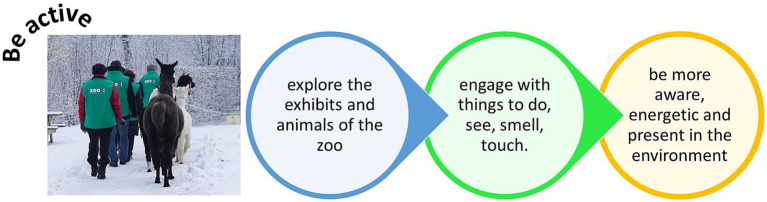
Being active at the zoo to improve physical health and how to connect with nature.

Any visit to a zoo or aquarium involves activity. Engaging in regular physical activity is beneficial for physical health and improves mental well-being (Warburton et al., 2006). And physical activity that is outdoors and embedded in nature alleviates stress and boosts quality of life during challenging periods of living (Egerer et al., 2022). Zoos can capitalize on such physical activity by outreach events and programs that can provide multiple opportunities for physical exercise, the broadening of social connections and chance to do or learn new things. Opportunities for engagement that encourage activity can include physical exercise, such as walking around exhibits and between enclosures, to engagement with educational activities that may involve creating, making, doing, or crafting (Figure 3). Walking tours and guided experiences also increase opportunities for physical activity around the zoo. These events are documented as being particularly effective at enhancing connectivity with nature from student groups (Kleespies et al., 2020) to middle-aged adults (Kleespies et al., 2022) and provide a way of promoting the intrinsic value of nature to urbanized audiences that may appear removed from biodiversity (de Lima, 2016). When an audience starts a tour with low nature connectivity, the event appears to be most effective at improving and increasing the individual’s sense of value of nature (Kleespies et al., 2020). Given the staple of guided tours around zoos, this form of physical activity, coupled with the potential for large influences in positive connections to nature, would be something for zoos to capitalize on and promote more widely.

Participation in guided tours with other visitors could develop new social bonds and a sense of connection with other likeminded individuals that have similar interests and passions. These events involve activity (walking around the zoo itself) but also could encourage activity away from the zoo, and opportunities to explore other green or blue spaces that the visitor learns about during the visit. For example, zoos may manage a nature reserve at a separate location; if these nature reserves are highlighted to participants on a tour, a new venue for physical activity is made available for people to potentially engage with. Guided tours increase participants knowledge and education on a specific topic (Whitehouse-Tedd et al., 2022) and as such, could be used to present other opportunities for activity that leads to increased connection with nature both at and away from the zoo. Such guided tours may be particularly important for people who visit a zoo alone to give a chance to strike up conversation with others around them and to therefore broaden their own social environment. Zoos provide multiple topics of conservation and moving between enclosures provides a variety of sensory experiences that can be discussed, explained, and explored. Opportunities for this activity could themed for a specific audience to encourage uptake on a tailored activity with key aims for that demographic.

Involvement in “keeper for a day” schemes or other opportunities to work with animals, such as volunteer programs, are also beneficial for improving visitors expectations and engagement with nature (Meadows, 2011; Ferguson and Litchfield, 2018). Such experiences add more opportunities to complete physical activity, to bond with others and to experience nature close-up. Whilst caring for zoo animals is physically demanding work, zoos should consider developing volunteer programs that are accessible (where logistically possible) to all sections of society and particularly consider outreach to less mobile individuals that would still benefit from close encounters with nature. Examples of widening participation in such experiences are found within the industry (Sydney Zoo, 2021; Blackpool Zoo, 2023) and this highlights the evolution of how zoos are encouraging the widest spectrum of society to come and engage with their messaging, key objectives, and with their living collections. As direct encounters with the animals themselves also involve physical activity, so this helps foster a connection with particular species in the zoo, and with nature more broadly.

Of course, zoos employ real keepers and a host of other staff who engage in physically demanding work as they clean animal enclosures, prepare animal diets, build or repair infrastructure and generally walk the many paths at the zoo as they visit different areas of the zoo as part of their work. Here another opportunity presents for zoos to evidence humans being active and fulfilling the second of the Five Ways for Wellbeing. In the sparse research into zookeeper opinions of their role and work environment, keepers acknowledge the ‘hard work’ their job entails; they describe ‘a calling’ and a need to work with animals, the importance and meaning of their role, but verbalize the sacrifice such physically and emotionally demanding work requires in terms of financial limitations, vigilance and the burdens of responsibility (Bunderson and Thompson, 2009). Thus, a need to further explore zookeeper wellbeing transpires as these are active people, engaging enthusiastically with the sensory riches that their site of employment affords yet such wellbeing benefits are potentially at risk from the burdens of responsibility zookeepers report experiencing.

## 4. Take notice

**Figure 4:**
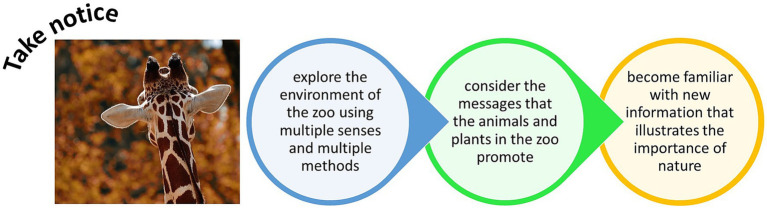
Taking notice at the zoo to learn about nature in new ways and feel more connected to the natural world.

Watching and experiencing the presence of zoo animals can encourage visitors to take notice of important messages (Grajal et al., 2017; Moss and Pavitt, 2019) that could enable personal growth and development. For example, by learning about previously unknown facts, concepts, and theories, or by developing pro-conservation Behavior and engaging with tools to become more sustainable. An integrated approach of signage and other forms of communication and interpretation (such as interactive engagement with social media platforms) has been shown as particularly effective at crystallizing key biodiversity messages to zoo visitors (Pearson et al., 2014). Zoos are places where people come to have encounters with other species (Rice et al., 2021a) and by seeking out such close encounters, visitors are taking more notice of the natural world and are being more connected to aspects of nature. By encouraging visitors to take notice, of the animals in the zoo and in a wider context, and of the visitor’s own learning and development, zoos can help people to reconnect with the wider world around them (Figure 4).

There is multiple evidence of how zoos contribute more widely to scientific outputs that can benefit wider society. Publication and dissemination of empirical science in the popular press and across media channels make scientific outputs more relevant and relatable to general, non-technical audiences (Farinella, 2018). Across zoo membership and accreditation organizations, zoo and aquarium research is answering new questions and providing insightful and impactful information on a wide range of subjects (Loh et al., 2018; Hosey et al., 2019; Hvilsom et al., 2020). Scientific outputs from zoos improve our theoretical and applied knowledge of the natural world (Conde et al., 2019; Rose et al., 2019) and such information can be presented in an accessible and engaging way to visitors to encourage uptake and memory of important messaging (e.g., around a species’ ecology or conservation or adaptations).

Zoos also engage in sensory experiences with their visitors that encourage people to take notice of their environment in different or extraordinary ways. For example, sound walks where visitors are encouraged to experience the zoo by listening to their environment and not through sight (Rice et al., 2021b). Sound walks are unlikely to be fully accessible to visitors with hearing impairments, but these activities can open up the zoo’s environment in new ways for people with other sensory disabilities, e.g., those that are visually impaired. By encouraging visitors to engage with different senses, a new perspective on the zoo, its animals and what it means to be in nature can develop.

Zoos can also offer mindfulness programs and activities centered around this mental health paradigm. Mindfulness refers to *“a moment-by-moment awareness of our thoughts, feelings, bodily sensations, and surrounding environment, through a gentle, nurturing lens”* (University of California Berkeley, 2023). And anyone practicing mindfulness is required to accept, and not judge, their current thoughts and feelings; to accept who they are in that current space and time. Mindfulness concepts can be built into wildlife encounters and experiences to promote a deeper connection with the natural environment and to enhance learning and engagement (Woods and Moscardo, 2003). The sound walks, as mentioned above, can encourage “acoustic mindfulness” and reflection on the lives of the non-human animals at the zoo (Rice et al., 2021b), therefore deepening participant’s connection with nature on a different sensory level. Visitors on mindfulness walks at the zoo can be encouraged to pay close attention to their surroundings, and notice the colors, shapes, sizes and activity patterns of the animals, and how the animal fits into its environment. Some zoos provide a guide and instructions on how to practice mindfulness on a zoo visit (Meek, 2016; Chester Zoo, 2023) including ideas for things to do (what to watch and experience), what to not do (e.g., avoid needing to photograph everything that can be seen or viewing the world via a mobile phone screen), and how to engage multiple senses.

Mindfulness is not simply a statement or singular reflection in time and space, it is a practice that is developed and fine-tuned with repetition and application; there is a level of dedication involved in focusing your thoughts on to your current state of being. Mindfulness refers to “observation without criticism” (Williams and Penman, 2011) allowing negative thoughts to be noticed before they have chance to fully infiltrate a person’s psychology. Therefore, zoos should work to embed mindfulness practice into a visit, offering opportunities for focused thought and the quiet reflective spaces needed to achieve a truly mindful state. Other forms of mindfulness out in nature, such as “forest therapy” result in numerous physical and psychological benefits to participants (Han et al., 2016; Rosa et al., 2021). Zoos should capitalize on such research to build and promote their own mindfulness programs, especially as many zoos are wooded and could participate in similar forest therapy style events. If zoos can provide such opportunities, both visitors and staff may reap the benefits, including reduced anxiety and depression (Khoury et al., 2015), lower pain scores (Reiner et al., 2013), and improved immunity (Davidson et al., 2003). For visitors, this increases the likelihood of returning to the zoo and valuing the zoo’s work. In turn, this creates longevity in the zoo’s appeal and its influence. For zookeepers, mindfulness events afford opportunities to deal with the burdens of responsibility that zookeeping entails. Zookeepers need time to reflect and focus their thoughts, allowing them to preserve their own wellbeing and better notice when the wellbeing of animals in their care changes and intervention is required. As such, as human wellbeing improved, so does animal wellbeing also improve.

## 5. Keep learning

An integral aim of the modern zoo is education (Kleiman, 1985), which adds value to the zoo’s living collection, its operations and impacts on society more widely (Greenwell et al., 2023). Zoos have well planned and structured educational offerings for pre-school, school, college, and university-level groups, and provide a wealth of informal educational materials and activities for general visitors too. The importance of zoo education programs is well reviewed and often evaluated to ensure efficacy (WAZA, 2023; EAZA, 2023b). Formal education sessions and informal educational activities can develop the participants’ connection with nature (Packer and Ballantyne, 2010; Kleespies et al., 2020, 2022). Zoo visitors are receptive to information on wider global issues, e.g., implications of climate change (Taylor and Duram, 2021), and as zoos can promote lifelong learning (Luebke et al., 2012), visits to the zoo can improve awareness and understanding of such global issues to promote positive Behavior change that benefits the quality of life of multiple individuals. As visitor attitudes and perceptions are influenced by the visual messages that they receive as they move through the zoo (Reade and Waran, 1996), zoos should consider the visitor’s journey through the zoo and how opportunities for learning are presented and made available at different enclosures and exhibits. Learning stations and interpretation also needs to consider the demographic at the zoo and perhaps ensure that adult visitors are catered for, as well as children.

Further development of how zoos use social media to provide information to their visitors should be undertaken to maximize integration of real world and online experiences. For example, research on use of social (e.g., a social media platform) and mobile (e.g., personal mobile phones) technologies as part of a museum visit revealed wider engagement of participants, provoked multiple opportunities for social exchange and did not interfere with real time engagement with the physical artifacts on display (Charitonos et al., 2012). These authors also note the importance of integrating social and mobile technologies into educational visits to encourage engagement with overlooked or disadvantaged groups of people.

**Figure 5:**
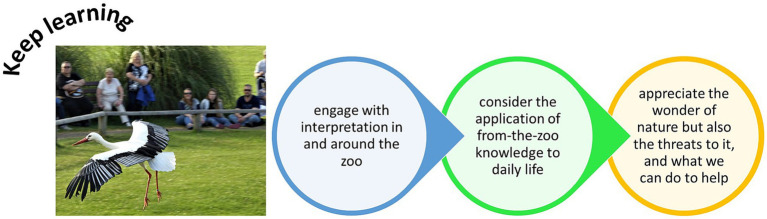
Opportunities to keep learning by visiting a zoo, interacting with signage and with animal displays or encounters, and taking away key information to promote positive human Behavior change.

Many zoos offer educational talks or presentations by zoo staff. Attending these sessions can provide valuable insights into animal Behavior, conservation actions, and the importance of biodiversity to human and planetary health (Figure 5). Live animal shows can be successful in connecting visitors to nature if they display the animal’s adaptations and natural Behavioral traits (Povey and Rios, 2002; Spooner et al., 2021), therefore informal education that connects the audience to the animal and its environment is achieved through the display of the animal’s evolutionary characteristics. Linking evolution to ecology, and then to threats and challenges that populations face (e.g., habitat loss and population reductions due to human activities) may allow an audience to see just why animals are threatened, because they possess specific traits and adaptations for specific environments that humans are destroying. Such sessions can grow each individual’s knowledge of conservation issues (Spooner et al., 2021) and, if such information is included in the demonstration, could become tangible tools that encourage audiences to be more sustainable and planetary friendly in daily life (Mellish et al., 2017).

Such sessions may also provide opportunities for social connections with others, and the chance to build links at the zoo that may result in longer term volunteering roles or similar. Using virtual reality (VR) to augment keeper presentations and educational sessions can bring the otherwise unseen day-to-day care of the zoo’s animals to the visitor’s attention (Carter et al., 2020). Employing novel technologies, such as VR, alongside of social media platforms or app-based methods could inspire deeper and more impactful learning at the zoo to a wider demographic. Visitors respond positively to the presence of VR alongside of also experiencing the live animal in the same space (Carter et al., 2020). Such integrated approaches, of presenting the animal in an enclosure, and of using other forms of technology to reduce distance between the animal and the visitor, could be employed (and evaluated) to see how well connection to nature is advanced, if information presented about the animal is retained for longer, and if visitors feel a deeper bond with the animal they are viewing.

When zoo staff prepare such formal and informal, active and passive learning opportunities, so to are they also experiences their own learning opportunities. Welfare, Behavior, conservation, research and animal care staff at the zoo must embed the latest scientific literature into practice, and therefore must engage with continuous professional development opportunities to ensure they can follow an evidence-based approach. Building mutually respectful and trusting collaborations with academic departments can enable access to scientific research papers and further opportunities for professional development (Fernandez and Timberlake, 2008; Schulz et al., 2022). Regional zoo associations also run CPD training events from across a broad spectrum of topics from general zoo governance to species-specific husbandry (ABWAK, 2023; BIAZA, 2023b). Zoo staff report valuing such learning opportunities but do not always feel supported to seek out or attend conferences and events (Bacon et al., 2021). Supporting zoo staff to attend such events is therefore essential because it allows learning to occur and further enhances staff social and professional connections across and within organizations. It also adds value to the diverse job roles at the zoo and allows staff opportunities for positive reflection on their own self-development.

## 6. Give

Being at the zoo also encourages people to give back to try and help the natural world in some capacity. Giving does not mean material items or financial donations, although (where this is financially able and fiscally responsible) donating money to a charity does improve mood (Geng et al., 2021). Those working at the zoo already give back, sometimes with limited financial reward or opportunity for career progression, as zookeepers report self-sacrifice while seeing they have a moral obligation to provide good welfare opportunities for the animals in their care (Bunderson and Thompson, 2009). The public too have opportunities to give. Volunteering time is a form of giving that zoos readily facilitate (BIAZA, 2023c). Research suggests that acts of giving help improve mental wellbeing by creating positive feelings and a sense of reward, promoting feelings of purpose and self-worth, and helping to establish connections with others in the community (Lum and Lightfoot, 2005; Rochester, 2006; Vannier et al., 2021). Although not all research agrees with ideas that volunteering always brings wellbeing benefits (Whillans et al., 2016), study of zoo volunteers shows a profoundly positive response to the work that they conduct (Fraser et al., 2009). Volunteers that are trained, and therefore feel invested in, can report the largest positive outputs from their work (Smith et al., 2018). Therefore, to ensure positive mental health outcomes, volunteer programs should align (as best possible) with the volunteer’s expectations, wants and needs from the work and any pre-existing skills and expertise. As well managed volunteer programs, that value and invest in their volunteers, can increase uptake of pro-environmental and pro-conservation Behaviors (Bixler et al., 2014), zoos can improve connection to nature and provide fulfilling and meaningful community engagement opportunities *via* their application of volunteers. Although the zoo and its operations will benefit from the presence of volunteers, it is essential that zoos see volunteers as more than this (Smith et al., 2018), and actively provide programs for development and learning alongside of the duties required of the voluntary position.

**Figure 6:**
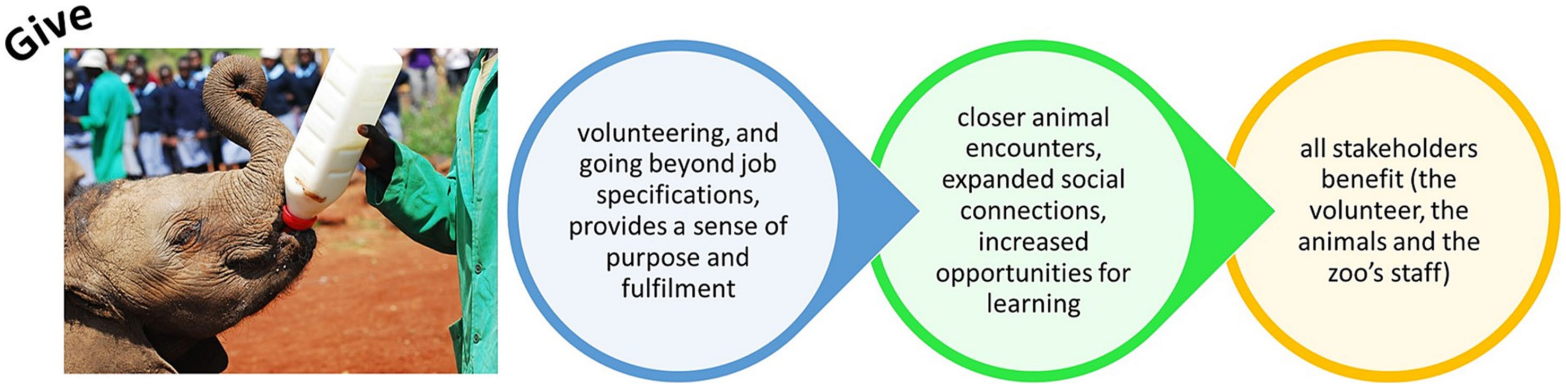
Providing opportunities for people to give back at the zoo can increase social capital (i.e., investment in friendships and in the activities of the organization) and increase human capital (i.e., personal knowledge and skills).

Volunteering increases human and social capital (Forbes and Zampelli, 2014). Human capital can be defined as the *“knowledge, skills, and health that people invest in and accumulate throughout their lives”* (The World Bank, 2022), and the extent of this capital helps realize an individual’s potential productivity to society. Social capital is harder to define but considers the social relations that individuals can form that have productive benefits (Institute for Social Capital, 2023), for example opportunities to form, develop and invest in professional and personal relationships that have meaning to the individuals involved. Zoos should consider human and social capital in terms of benefits to the volunteer and to the organization, and to nature conservation and planetary health more widely, when designing and implementing volunteer schemes. The volunteer giving time to the zoo, and the zoo giving resources and opportunities to the volunteer strengthens the overall impact of this relationship on the zoo’s education, engagement and conservation aims, and can boost the positive quality of life outcomes experienced by the individual who is volunteering (Figure 6). This of course is also the case for those employed at the zoo and who go above and beyond to uphold and evidence industry values, the zoo’s mission statement and public expectation relating to animal care. Further research into the personal goals and aspirations of volunteers, their motivations behind taking on the role, what they have gained from it and why they feel this is important should be conducted more widely. Such research would provide evidence for how to develop volunteers, maintain their interest and enthusiasm, and ensure they feel valued and appreciated.

## 7. Discussion

In this article we have shown that working at or visiting a zoo enables connection with biodiversity and positive feelings of wellbeing. We suggest that zoos can consider a Five Ways to Wellbeing approach when discussing and implementing their living collection plans, designing and crafting visions, mission statements and operational strategies, and embedding opportunities for formal and informal learning for all visitors, staff and volunteers. Each of the Five Ways to Wellbeing are not mutually exclusive. Engagement with one leads to involvement in activities that fulfill many of the others. The multi-dimensional nature of a zoo visit (from seeing the animals, to engaging with people, to exploring a new environment, to the travel to and around the facility, to learning new information and developing ways of using such information) provides a unique way for visitors to become immersed in an environment that can positively impact on physical and mental health. Zoos need to ensure that the environment presented to visitors is a positive one. Animals need to be healthy, behaving in a species-typical, ecologically relevant manner, and all signage, interpretation and messaging needs to be clear and transparent. Zoos educational and conservation messages can be diluted if animals are behaving abnormally and if visitors leave with a poor view of how seriously the zoo views animal welfare. Therefore, developing the zoo as tool to improve mental health and human wellbeing goes hand-in-hand with developments to animal husbandry and management. The zoo must keep abreast of scientific evidence for best practice husbandry (Rose, 2018) and continue to enhance and evolve enclosures and exhibit design so that animal welfare is also good and all living beings maintained in the zoo’s collection have an opportunity to experience “a good life” (Green and Mellor, 2011).

Monitoring of physical and psychological outputs during a zoo visit show that the activity of walking around a zoo reduces blood pressure, increases step rates and improve positive outlooks on life (Sakagami and Ohta, 2010). Encouraging activity by taking visitors on a journey around different enclosures and exhibits therefore has multiple health and wellbeing benefits, as well as encouraging people to feel more relaxed and, therefore, potentially keener to be in the moment and connect to nature. The New Economics Forum has evidenced that those who have strong social relationships, are physically active and continue to be involved in learning experience improvements to wellbeing and physical health (Aked et al., 2008a), as social bonds, an active lifestyle and opportunities for learning are all important influencers of health and wellbeing. If zoos are able to identify wellbeing needs in their local communities and target reduced price visitation accordingly, the Five Ways to Wellbeing could be more readily realized for more people who are disconnected from nature. Using the zoo as a way to exercise, whilst learning for example, may open up further possibilities to engage with different demographics.

Being active in the zoo can help people to “move their mood” (Tonkin and Whitaker, 2021) and spending time on leisure activities at the zoo (with family and friends) can instigate conservations, discussions and dialog to help people feel more connected. Research has identified that spending time in immersive zoological exhibits improve the mood of visitors (with self-reported feelings of happiness increasing) and reduces stress (Coolman et al., 2020). Zoos should build on these positive findings by providing maps, trials or tools that relate to the Five Ways to Wellbeing to easily share this concept with zoo visitors. Not all mental health challenges are discussed or visible, and a lowkey approach to improving mood and emotion may help individuals, when they leave the zoo, make changes to their daily lives that will improve their quality of life and reduce anxiety.

For those immersed in these environments as their place of work, there are connection benefits too, particularly for animal care staff. Their roles require undertaking physically demanding work yet their willingness to “give back” beyond their job description resonates as they see the value in their efforts to animal welfare and conservation outcomes. Compassion fatigue is a genuine risk to animal care staff (Figley and Roop, 2006) as the toll of seeing animals failing to thrive can manifest into acquiescence. The relevance of good animal welfare here is paramount – seeing animals thrive brings a sense of proud fulfillment and pride in one’s job. This enhances happiness and creates opportunities to connect with other staff to share successes and good practice, and consequential scope for mindful happiness. Zoos should value their staff and sufficiently support their needs, both personal and professional, while prioritizing animal welfare to evidence the Five Ways to Wellbeing in their extensive workforce.

Key challenges that zoos face to provide a more egalitarian “Five Ways to Wellbeing” experience centre around entry costs and accessibility. Collaboration between institutions when concerning supplies, logistics and procurement, could reduce operating costs (Baptista et al., 2021) and therefore zoos may be able to make reductions to ticket prices for low income groups or for sections of society that may have less disposable income to expend on entry tickets. Corporate sponsorship of reduced ticket entry could widen access to the zoo, and zoos should continue to build relationships with industry partners that could help subsidize ticket costs for key demographics that zoos wish to engage with. Zoos should consider the impact of ticket pricing as a potential barrier to engagement with their work, and engage with external social initiatives, widening participation schemes and philanthropy within their local community to attract audiences that may be unable or unwilling to visit. For example, the “Generation Wild” initiative at the Wildfowl & Wetlands Trust (WWT) aims to break down barriers to access to nature, and provides free site entry plus follow-up learning opportunities to build pro-nature, pro-conservation attitudes and Behavior change in adults and children alike (WWT, 2023). Increasing the use of different tools for communication could provide zoos with a way of reaching a wider societal demographic with associated wider societal impacts. Multiple layers of interpretation have been shown as the most effective way of instilling memorable and relatable messaging when experiencing a zoo exhibit (Weiler and Smith, 2009). Therefore, combining different media and formats of messaging could help zoos extend the reach of their key educational outputs and encourage more people to feel connected to nature, as well as encouraging the Keep Learning aspect of the Five Ways to Wellbeing.

Zoos also need to consider how disconnected visitors, volunteers and staff may initially be from nature. Oh et al. (2021) demonstrate that spending a longer in nature than usual can be more stressful or anxiety-inducing if the person’s baseline connectivity to nature was weak to begin with. Therefore, zoos need to be mindful of the background of individuals who they attempt to engage with, their prior experiences of the natural world and how they perceive any relationship with nature, before embarking on nature connectivity programs or events. In this article, we have provided an overview of the activities of the modern zoo that support these Five Ways to Wellbeing, in the hope that others will take these concepts, apply and test them to encourage new and effective ways of human engagement with the zoo’s mission and objectives.

Nature-based interventions within the zoo can be of benefit for specific groups of people. For example, individuals with disabilities are less likely to spend time in nature than able-bodied people (Sahlin et al., 2019). Providing nature based interventions for disabled people and their carers has positive educational outputs and improved caretakers enthusiasm for their profession by facilitating new ways of managing stress and providing tools to improve mood (Sahlin et al., 2019). Zoos should work on their outreach programs with under-represented groups, and those with limited access to nature, to ensure the zoo’s green and blue spaces, and the animal collection, are accessible to all those who may benefit from being immersed in a natural setting. Investing in such initiatives and objectives today means ensuring visitor footfall and recruitment of a sustainable workforce in the future.

Zoos should continue to research the potential of their living collections as being beneficial to nature connectivity and as a tool to improve emotions and mood. Research identifies that whilst there can be common, positive findings on how observing and interacting with animals improves human wellbeing (Sahrmann et al., 2016; Gee et al., 2019), methodological limitations, biases in experimental design and lack of repeatability can reduce generalisability of research findings (Clements et al., 2019). Cross institutional research, using standardized methods and pre-registering projects to encourage scrutiny prior to data collection may help to generate more robust conclusions that can help decipher exactly why being in nature, or being near animals, is beneficial to human wellbeing.

Zoos can increase connection to nature by considering the situation that experiences take place in. Research on situational interest, i.e., the specific features of a place, location or artifact (Schiefele, 2009), can provide zoos with information on how to present learning opportunities to increase connection with nature. A zoo’s landscape ecology, it’s “zooscape” (Bisgrove, 2022), can promote connection to nature whilst explain the ecological and social importance of habitats and green/blue spaces. Bonderup Dohn (2011) found that school children who were presented with learning activities within an aquarium responded positively to the experience because of the setting. This research identified that the children’s social involvement, the hands-on element of the activity, the activity being a surprise and novel, and the aspect of knowledge acquisition as the main outcome, to be key triggers of interest. These findings are useful for zoos to consider when planning and designing both formal and informal education sessions and when they wish to foster the interest of their visitors in important, fundamental topics (e.g., biodiversity conservation).

Whilst zoos are working hard to expand their wider influences and extend their role in society, there are still areas of publication output and scientific enquiry that can be worked on Rose et al. (2019). For example, Anzai et al. (2022) shows that not all zoos can have a focus on scientific research and not all research enquiry focuses on the key aims of the modern zoo. Therefore, zoos should continue to increase collaboration and the development of relationships across their own industry and externally too (e.g., with academic departments at universities) to enable all important aspects of their operations to be evidence based. Ultimately, zoos and aquariums need to place a greater emphasis on animal welfare and on human wellbeing as part of their core aims, operational outputs and influence over human populations (their visitors, staff and stakeholders). As examined by Rose and Riley (2022), cementing Wellbeing as a key aim of the modern zoo provides clear evidence to all invested parties that zoos fundamentally care about animal welfare and human wellbeing because they are working to promote, protect and preserve positive aspects of mental health.

Research has identified that people who care about threats to the natural world are more likely to spend time at the zoo and view the zoo’s work as positive for nature conservation and as a way of encouraging planetary friendly Behavior change (Taylor and Duram, 2021). However, there are many people who may not consider visiting the zoo (as a way of interacting with green and blue spaces) and so zoos should focus some of their efforts and resources on reaching groups of people that are less regular visitors or who never visit. For some, zoos can be controversial institutions whose aims appear contradictory (Wickins-Dražilová, 2006; Carr and Cohen, 2011; Maynard, 2018). Therefore, the idea of connecting with the natural world in the unnatural setting of zoo’s enclosures and exhibits may appear incompatible. Zoos should therefore promote and explain examples of Behavioral consistency between wild individual and those under human care. For example, parity of vigilance activity in meerkats that, even after many generations in captivity, still perform key wild-type Behaviors (Huels and Stoeger, 2022). This would demonstrate the care that zoos place in their husbandry and management to ensure that species in the living collections remain a true representation of nature. Zoos should also consider the language they use and how they promote themselves. For example, using the term “habitat” for an animal’s enclosure (Bruno et al., 2023) could be seen as disingenuous; a habitat is a biological system, where a species interacts with a myriad of biotic and abiotic interactions (European Environment Agency, 2023). A zoo’s managed environment controls these interactions, and therefore explaining to visitors how specific aspects and resources of a habitat are replicated within an enclosure may be a more honest way of educating visitors on species’ ecology. There is clearly a role for zoos in the protection, promotion and conservation of species that is promoted *via* public education (Whitehead, 1995; McCubbin, 2022). Getting the messaging right, being honest and transparent, and accessible, to encourage wider buy-in of such roles will enhance the relevance of the zoo to a wider demographic.

This article has explored the concept of the Five Ways to Wellbeing regarding the activities and operations of zoos that could be directly co-opted to promote human wellbeing and connection to nature. We have reviewed the scientific literature and practical examples of zoos’ works to demonstrate how the aims and goals of such activities can improve human health and wellbeing, promote access to green/blue spaces and support more opportunities for nature connectivity. Due to the nature of a review paper, we are unable to evaluate or analyze the timescale, logistical considerations, financial requirements, or personnel needs of successfully embedding the Five Ways to Wellbeing into the work of the modern zoo. Empirical information is required to understand how well our suggested Five Ways to Wellbeing concepts and approaches would fare in practice. Further research into the design of a Five Ways to Wellbeing initiative or activity, followed by its implementation, and eventual evaluation and assessment of measurable impact is required to fully evidence the relevance of this approach to the positive outcomes of visiting a zoo and engaging with its living collection and green/blue spaces.

## 8. Conclusion

This article has reviewed how a Government-instigated initiative that aims to improve human mental health and quality of life could be useful for zoological collections to consider as a way of working to enhance the wellbeing of their communities and improve connection with nature. Our article shows how the key concepts of the Five Ways to Wellbeing can form a framework for zoos to further engage with their human audiences. Each of the Five Ways to Wellbeing is relevant to the work that zoological collections do for their staff, visitors, and the wider communities around them. We have shown that zoos contain many useful and relevant exhibits (e.g., animals within their enclosures), programs (e.g., educational activities and public talks), and resources (e.g., open green spaces, planting, biological artifacts) that together provide multiple opportunities to apply the ideas of Five Ways to Wellbeing. The zoo’s most important resources is its living collection of plants and animals; by tailoring the use of the living collection to improve engagement with the natural world and to better connect their workforce and visitors to nature, zoos are not only able to advance wellbeing of their human stakeholders but also add more value to that already intrinsic within the living collection itself.

## Author contributions

PR: Conceptualization, Writing – original draft, Writing – review & editing. LR: Conceptualization, Writing – review & editing.

## Footnotes

^1^ scholar.google.com

^2^ webofscience.com
